# Exploring neural mechanisms of gender differences in bodily emotion recognition: a time-frequency analysis and network analysis study

**DOI:** 10.3389/fnins.2024.1499084

**Published:** 2024-12-17

**Authors:** Tingwei Feng, Mingdi Mi, Danyang Li, Buyao Wang, Xufeng Liu

**Affiliations:** ^1^Department of Military Medical Psychology, Fourth Military Medical University, Xi’an, China; ^2^Weinan Vocational and Technical College Student Office, Weinan, China; ^3^College of Education Science, Changji University, Changji, China; ^4^Mental Health Education and Consultation Center, Tarim University, Alaer, China

**Keywords:** physical emotions, time-frequency, source analysis, network analysis, time domain

## Abstract

**Background:**

This study aimed to explore the neural mechanisms underlying gender differences in recognizing emotional expressions conveyed through body language. Utilizing electroencephalogram (EEG) recordings, we examined the impact of gender on neural responses through time-frequency analysis and network analysis to uncover gender disparities in bodily emotion recognition.

**Methods:**

The study included 34 participants, consisting of 18 males and 16 females. A 2 × 2 mixed design was employed, with gender (male and female) and bodily emotion (happy and sad) as the independent variables. Both behavioral and EEG data were collected simultaneously.

**Results:**

Males demonstrated more stable brain activity patterns when recognizing different bodily emotions, while females showed more intricate and highly interconnected brain activity networks, especially when identifying negative emotions like sadness. Differences based on gender were also observed in the significance of brain regions; males had greater importance in central brain areas, whereas females exhibited higher significance in the parietal lobe.

**Conclusion:**

Gender differences do influence the recognition of bodily emotions to some extent. The primary aim of this study was to explore the neural mechanisms underlying gender differences in bodily emotion recognition, with a particular focus on time-frequency analysis and network analysis based on electroencephalogram (EEG) recordings. By elucidating the role of gender in cognitive development, this study contributes to early detection and intervention.

## Background

Emotion recognition is vital for social interaction and emotional expression, involving the perception and interpretation of emotional signals such as facial expressions, vocal tones, and body language (Rosenberg et al., 2020). This skill is essential for successful daily interactions and has significant clinical and psychosocial applications. Studies suggest that recognizing emotions through bodily postures is as accurate as through facial expressions in adults (Atkinson et al., 2004). Zongbao et al. (2019) used videos depicting facial and bodily expressions of anger, fear, and joy and applied multivariate pattern classification analysis of functional connectivity patterns using fMRI. The findings revealed distinct neural network representations for facial and bodily emotions of the same valence, with facial expressions achieving higher classification accuracy (Atkinson et al., 2007). However, bodily cues can sometimes convey emotional valence more effectively, especially under intense emotional states, due to the larger distinguishable area and greater distance compared to facial expressions (Zieber et al., 2014). Furthermore, individuals can accurately recognize emotions from brief presentations of dynamic and static bodily stimuli, even without facial information. These studies demonstrate that humans can effectively extract emotional information from bodily postural cues (Aviezer et al., 2012).

Network analysis is a powerful visualization model that represents complex relationships through nodes and edges (Borsboom and Cramer, 2013). This method is extensively used in psychometrics and clinical medicine to explore the interconnections and intrinsic relationships between psychological variables or symptoms (Epskamp et al., 2012; Epskamp et al., 2018). In this study, we apply network analysis to investigate the neural mechanisms underlying gender differences in bodily emotion recognition. By constructing network models, we can quantitatively assess the core positions of different brain regions, known as centrality indices, to determine their importance within the entire network. This approach allows us to identify key brain regions involved in emotion recognition and how they differ between genders. Additionally, network analysis helps reveal “bridge symptoms,” which are symptoms that connect different mental disorders (Liang et al., 2022). Applying this concept, we aim to uncover how different neural networks interact during the recognition of bodily emotions, providing deeper insights into the gender-specific neural mechanisms involved in emotional processing.

The objective of this study is to explore the impact of gender differences on bodily emotion recognition, providing additional insights into the neural basis of these differences. By employing high temporal-resolution EEG combined with time-frequency analysis and network analysis techniques, the research investigates whether there are gender-based differences in neural responses during bodily emotion recognition tasks and whether these differences reflect unique neural mechanisms related to gender in emotion processing. Time-frequency analysis offers the advantage of capturing dynamic changes in brain activity over time, although it may require more complex data interpretation compared to other methods. Additionally, network analysis provides a comprehensive view of the intricate connectivity patterns in the brain, allowing for the identification of how different brain regions interact during emotion recognition. This approach not only enhances the understanding of the underlying neural mechanisms but also highlights potential gender-specific network characteristics that could inform targeted interventions.

## Methods

### Participants

A total of 36 undergraduate and graduate students from the Air Force Medical University were recruited for the study, including 18 males, aged between 18 and 25 years, with an average age of 23.65 years. All participants were right-handed, had normal or corrected vision, and had no history of cognitive or neurological disorders. The study was approved by the Clinical Trial Ethics Committee of the First Affiliated Hospital of the Air Force Medical University (approval number: KY20182047-F-1). Written informed consent was obtained from all participants prior to the experiment, and the study adhered to the principles of the Declaration of Helsinki.

### Material

In this experiment, we used a 2 × 2 mixed-participation factorial design. The first factor was the trial condition, divided by gender (male vs. female), while the second factor related to the emotional expression displayed by the body, specifically happiness or sadness. The stimuli included 120 images obtained from the Chinese Library of Physical Emotional Materials (Luo et al., 2012), with valence and intensity rated on a 9-point Likert scale (Luo et al., 2010).

### EEG recording and data analysis

The raw EEG data were preprocessed offline using EEGLAB (Nagabhushan Kalburgi et al., 2024) for analysis. The EEG equipment used consisted of a NeuroScan SynAmps 2 amplifier and acquisition system, along with a NeuroScan 32-channel Ag/AgCl electrode cap for EEG recording. The study followed the international standard 10–20 system for electrode placement, with the left mastoid (A1) as the reference electrode and the central forehead as the ground electrode (GND), according to the guidelines of the International Society of Psychophysiology and the International Federation of Clinical Neurophysiology. After re-referencing, the reference electrodes were averaged from the left and right mastoids (A1, A2). The recording electrodes included FP1, FP2, F3, F4, F7, F8, Fz, FC3, FC4, FT7, FT8, FCz, T3, T4, C7, C8, Cz, CP3, CP4, TP7, TP8, CPz, P3, P4, T5, T6, Pz, O1, O2, Oz, and vertical (VEOG) and horizontal (HEOG) electrooculograms. During the experiment, the scalp impedance at each electrode site was kept below 5 kΩ, and the sampling rate was 1,000 Hz.

The preprocessing steps were as follows: data were analyzed using EEGLAB 14.0 software. After importing the data, electrode locations were verified, and unnecessary electrodes were removed. Re-referencing was performed with the average reference of the left and right mastoids (A1, A2). A 40 Hz low-pass filter and a 0.1 Hz high-pass filter were applied, followed by the removal of 50 Hz power-line interference. The data were segmented from −200 ms to 1,000 ms and baseline corrected. Bad segments were removed and bad channels interpolated. Independent component analysis (ICA) was applied to remove eye artifacts (HEOG, VEOG), ECG, and EMG artifacts. The final step was to average the segments.

The preprocessed raw EEG signals were analyzed using Fourier Transform to compute the power spectral density (PSD), with the power spectrum (in μV^2^/Hz) for each frequency range. After preprocessing, the EEG power was divided into six frequency bands: *δ* (1–3 Hz), *θ* (4–7 Hz), *α* (8–13 Hz), *β*1 (14–20 Hz), *β*2 (21–30 Hz), and *γ* (31–40 Hz).

Source localization was conducted to identify cortical regions involved in processing emotional stimuli. The sLORETA software was used to analyze the preprocessed data. A forward model was constructed based on individual head models, and an inverse solution was applied to estimate neural sources from the observed EEG signals. Statistical analysis of the resultant source activations was performed to identify cortical areas significantly associated with the different experimental conditions. Given the low spatial resolution and potential volume conduction issues inherent in source analysis, particularly in locating deeper brain structures such as the amygdala involved in emotional processing, the results were interpreted with these limitations in mind.

### Network analysis

Network analysis was performed using R (version 4.3.1) for network data analysis and visualization. In the network, 14 nodes represent different dimensions of behavioral and EEG indicators across various community subgroups. Data fitting was performed using the Gaussian graphical model (GGM), where edges (connections between two nodes) represent partial correlations. Model selection was conducted using the graphical lasso algorithm (least absolute shrinkage and selection operator, LASSO) combined with the extended Bayesian Information Criterion (EBIC) to obtain a more stable and interpretable regularized partial correlation network. The Fruchterman–Reingold algorithm was used to display the network layout. Network construction and visualization were implemented using the qgraph package in R. In the network, positive correlations are represented by blue edges, and negative correlations by red edges (Fruchterman and Reingold, 1991).

The thicker the edge, the stronger the association between two symptoms/variables, and vice versa. The expected influence evaluates the importance of each node within the network. Predictability of a node refers to the degree to which changes in the node can be predicted and explained by the changes in its connected nodes. In the network, predictability is estimated by the upper bound of its connected nodes’ predictions, represented by a surrounding ring around the node. The accuracy and stability of the network were assessed using the bootnet package. First, non-parametric bootstrapping (2,000 bootstraps) was used to calculate the 95% confidence intervals for edge weights to measure their accuracy. Second, case-dropping bootstrap methods were used to obtain stability coefficients for correlation, thus evaluating the stability of expected influence for each node. Ideally, the stability coefficient should be greater than 0.5, not lower than 0.25. Finally, bootstrap (2,000 bootstraps) was used to conduct difference tests on edge weights and nodes’ expected influences to assess whether the differences between edge weights or expected influences of nodes were statistically significant (significance level *α* = 0.05).

## Results

### Frequency domain analyses

**Figure 1:**
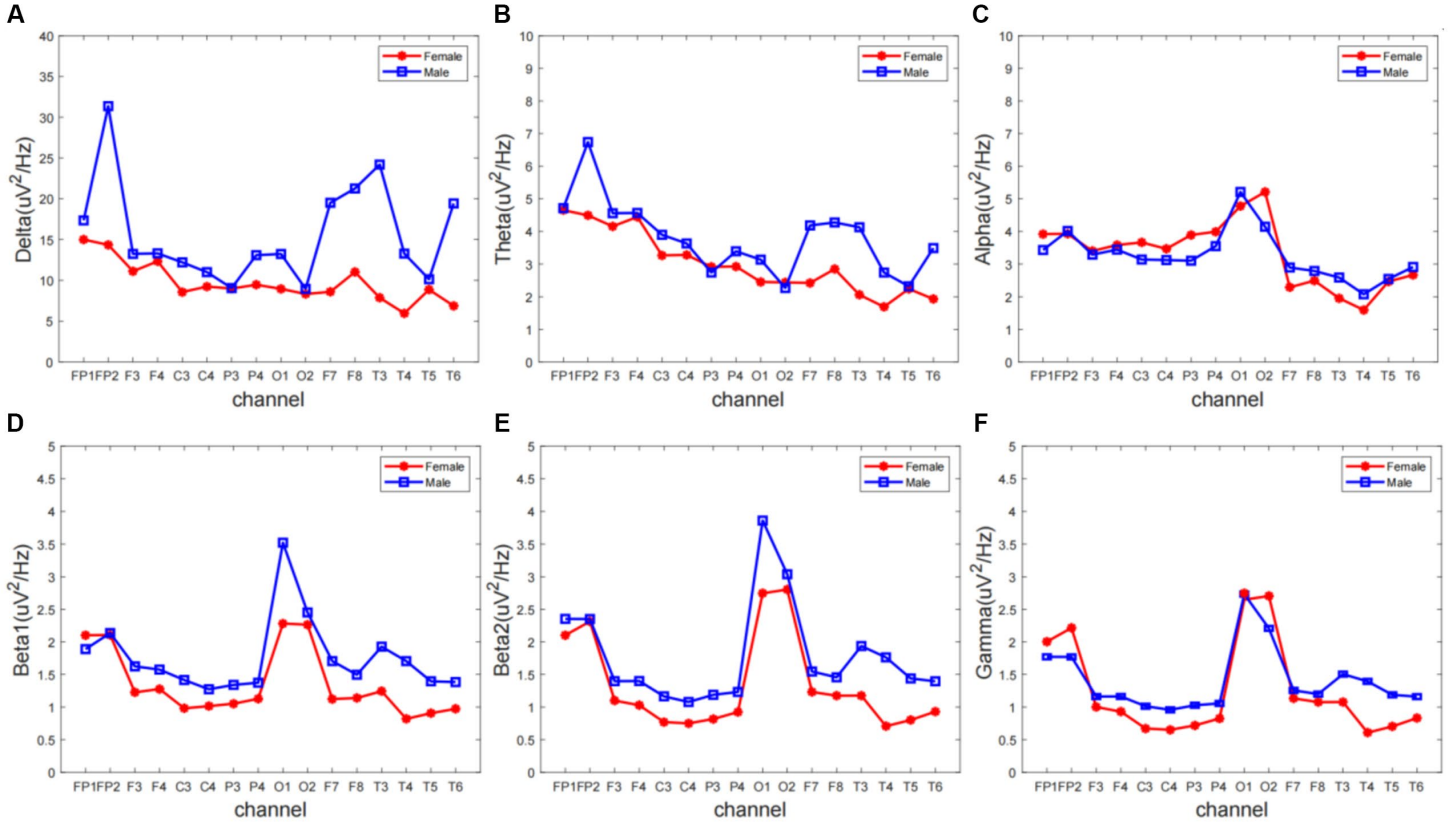
The overall relative power spectrum density (PSD) of 16 EEG channels in the **(A)** delta, **(B)** theta, **(C)** alpha, **(D)** beta1, **(E)** beta2, and **(F)** gamma.

We extracted ERP signals from a time window (−500 to 0 ms) relative to the stimulus onset, For each subject and electrode, EEG signals were transformed to the frequency domain using a discrete Fourier transform, yielding an EEG spectrum ranging from 1 to 30 Hz. Single-subject EEG spectra were averaged across subjects in each group, to obtain group-level prestimulus EEG spectra. To compare the group difference of prestimulus EEG spectra, we performed point-by-point independent-sample *t*-tests (i.e., each frequency point) for each electrode, with a false discovery rate (FDR) procedure. EEG contains different specific frequency bands. Features in sub-bands are particularly important to characterize different brain states. The sub-bands of interest are: delta (0.5–4 Hz), theta (4–7 Hz), alpha (8–13 Hz), beta1 (14–20 Hz), beta2 (20–30 Hz). The relative PSD can be obtained by dividing the PSD of each frequency band by the total PSD of the whole frequency band estimated by the AR Burg method. Figure 1 shows that the wave amplitude of the 16 EEG channels in the five frequency bands decreases with increasing frequency for both groups according to gender.

Different frequency bands of the electroencephalogram (EEG) have distinct physiological significance and are crucial for characterizing brain activity. To gain a deeper understanding of the rhythmic characteristics of EEG across frequency bands, this experiment averaged the power spectral density (PSD) values of the frequency signals from 16 electrodes of interest (FP1, FP2, F3, F4, C3, C4, P3, P4, O1, O2, F7, F8, T3, T4, T5, T6) across 5 different frequency bands. Repeated measures analysis of variance (ANOVA) was used for statistical analysis between the two groups.

To gain insight into the variation in the overall brain region frequency domain FFT, we further averaged the FFT values of 16 electrodes in six frequency bands. Statistical analysis was carried out using both traditional ANOVA and data-driven point-to-point *t*-test respectively, as shown in Table 1.

**Table 1:** Results of ANOVA for relative PSD of six frequency bands between female and the male.

Sub-band	*F*-value	*p*-value
Delta	−3.58	0.00^***^
Theta	−2.25	0.03^*^
Alpha	0.11	0.91
Beta1	−2.29	0.02^*^
Beta2	−1.71	0.09
Gamma	−1.22	0.28

^*^p < 0.05 and ^***^p < 0.001.

**Figure 2:**
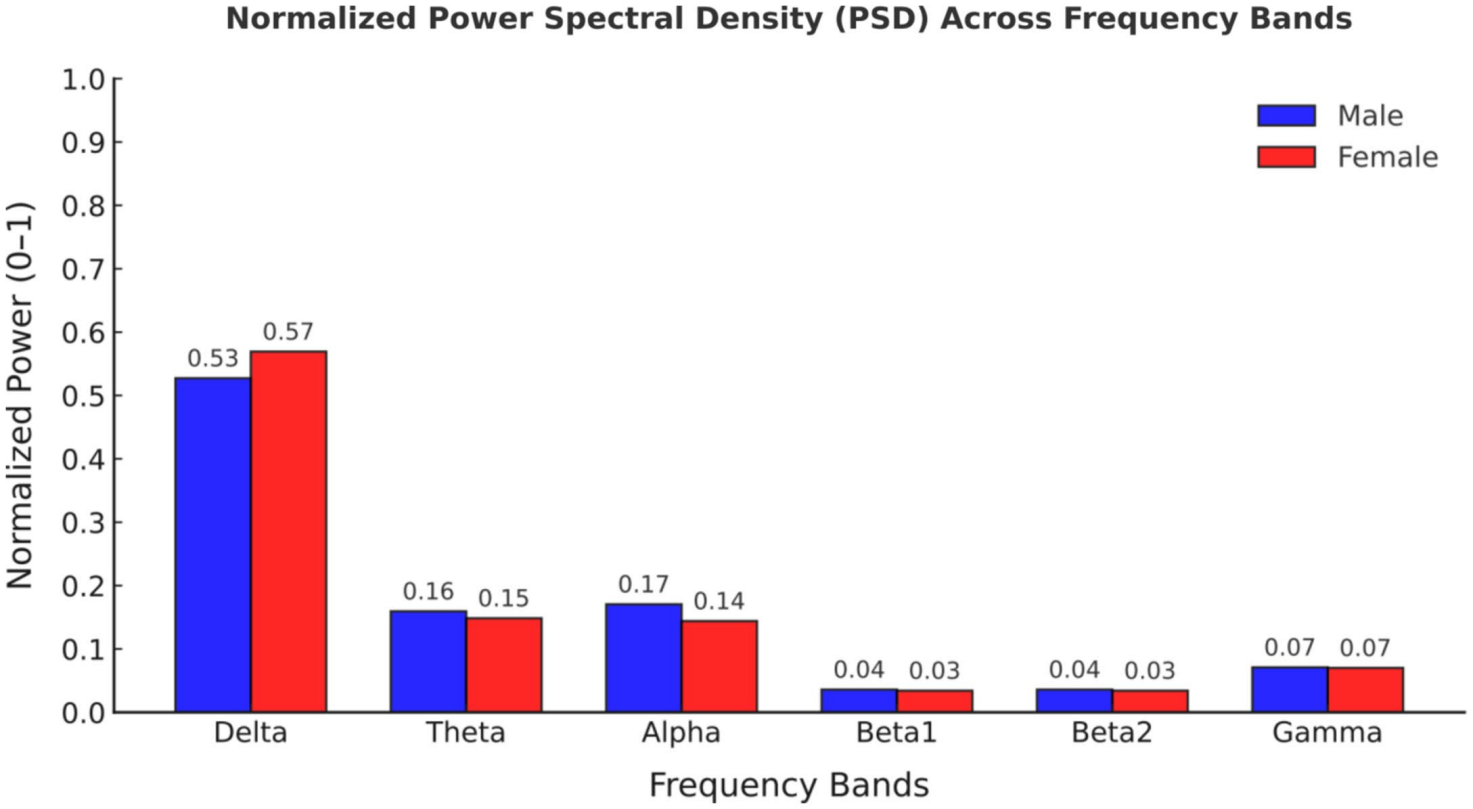
Normalized power spectral density (PSD) values across frequency bands for male and female participants.

Comparing the magnitudes in the frequency domain for the different gender groups, we obtained the following interesting results. Significant differences in FFT amplitude between gender groups in delta, theta, beta1 band. As shown in Figure 2, FFT amplitude in three frequency bands male > female.

### Time-frequency domain analyses

**Figure 3:**
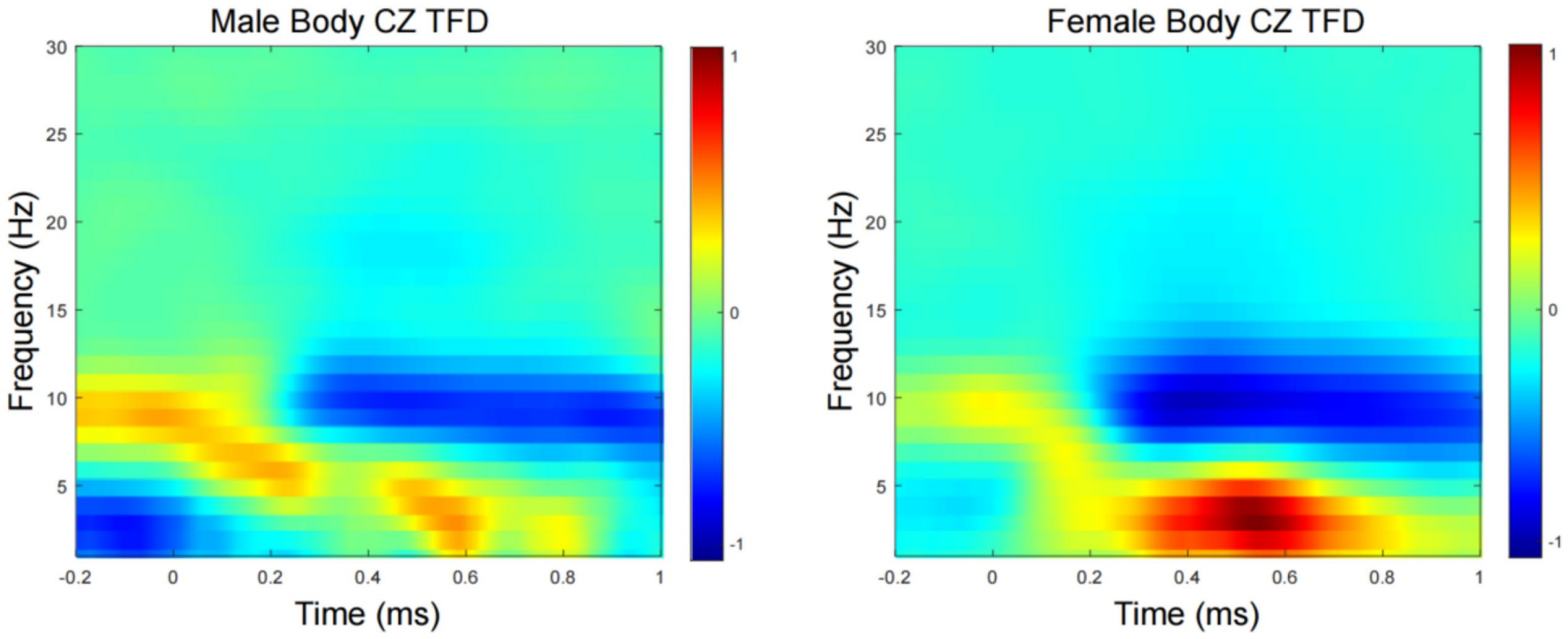
Time-frequency domain (TFD) of CZ channels induced power. Grand average TFD for the male and female conditions.

Figure 3 show the grand average TFD of evoked power for the male- and female-body emotion conditions. The TFD of the two conditions differed significantly in one cluster exhibiting larger power in the female- than male- body emotion conditions (*p* = 0.03), which was detected in theta-band (around 4–7 Hz) in the time window of around 400–600 ms after stimulus onset, mainly distributed over the CZ channels.

### Source analysis

**Figure 4:**
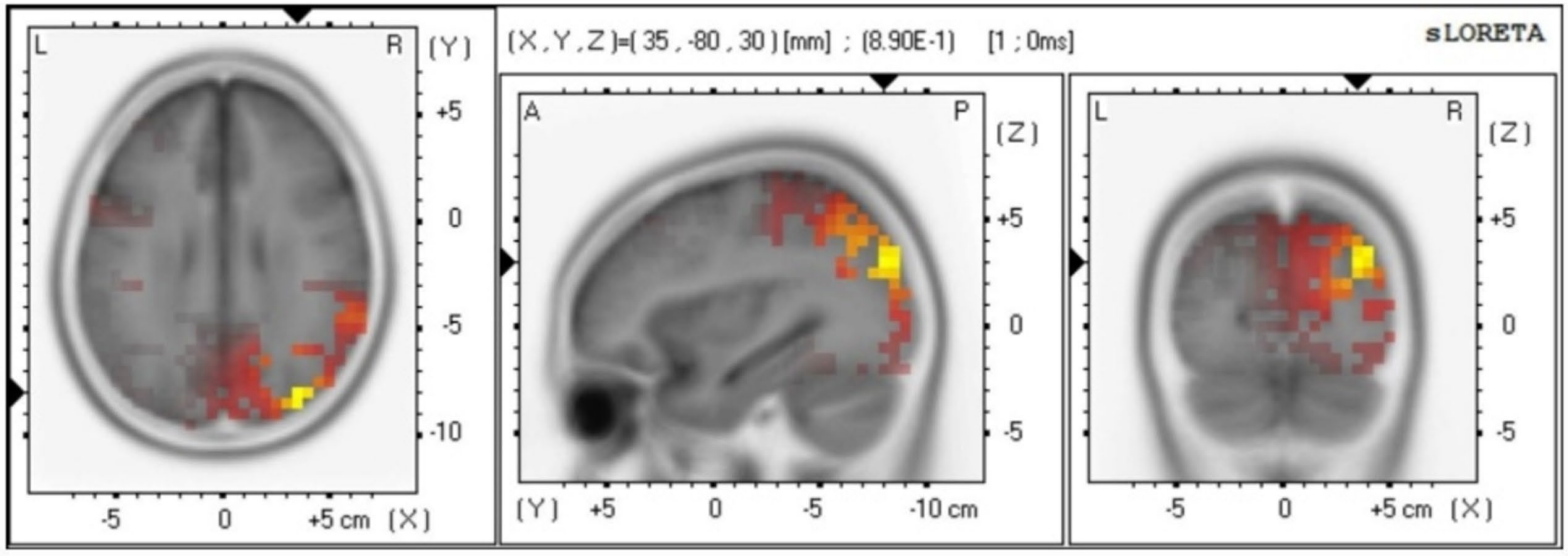
Gender differences in source localization analysis.

The source localization results indicate that the main differences between males and females are observed in the Broadmann area (Pirau and Lui, 2023; Peng et al., 2018), Lobe, and brain structure at 816–820 ms. In particular, Broadmann area 6 and 8 are responsible for integrating sensory information, memory, and regulating attention. Broadmann area 9 is primarily involved in speech motor control, coordination between limbs, and motor learning. Broadmann area 10 plays a role in processing stimuli (see Figure 4).

### Network analysis

**Figure 5:**
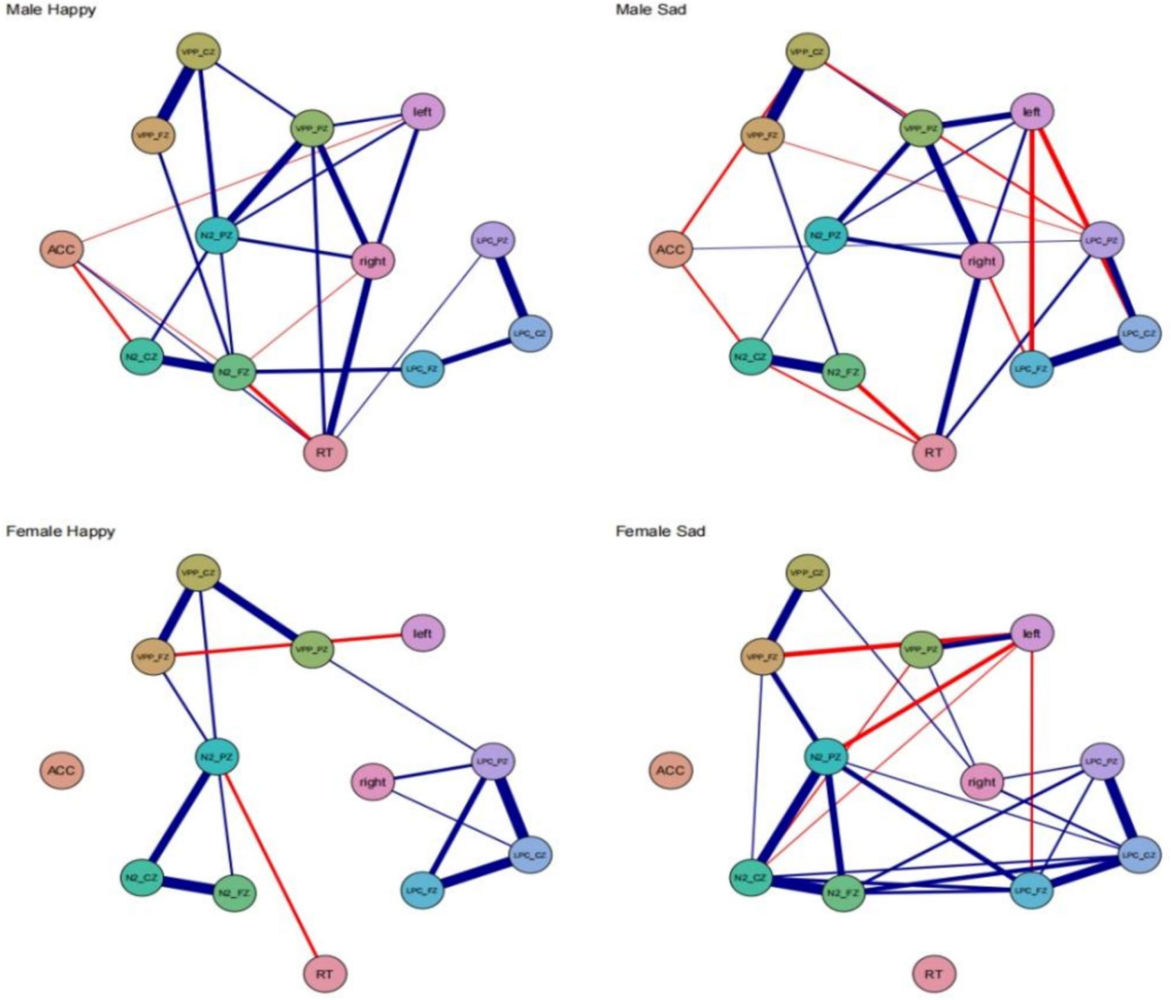
Network models for different gender groups of men and women of the network model and the ERP indicator level network.

Male happy the behavioral-electroencephalogram network structure, as depicted in Figure 5, shows the strongest edge between VPP_FZ and VPP_CZ (0.94). The N2_PZ node has the highest expected influence centrality at 1.49, while ACC has the lowest expected influence centrality at −2.70. ACC exhibits negative correlations with three ERP indicators: N2_CZ (−0.50), left (−0.41), and N2_FZ (−0.40). There is also a negative correlation between RT and N2_FZ (−0.55). Female happy the strongest edge is between N2_FZ and N2_CZ (0.92). N2_PZ has the highest expected influence centrality at 1.70, and this centrality is higher in females compared to males. In the female happy condition’s behavioral-EEG network structure, there are only two negative correlations: VPP_FZ-left (−0.58) and RT-N2_PZ (−0.51). Male sad the strongest edge is between VPP_FZ and VPP_CZ (0.87). LPC_CZ has the highest expected influence centrality at 1.48, while ACC has the lowest expected influence centrality at −1.90. ACC exhibits negative correlations with two ERP indicators: N2_CZ (−0.46) and VPP_CZ (−0.48). RT shows negative correlations with two ERP indicators: N2_FZ (−0.55) and N2_CZ (−0.45). The left node also exhibits negative correlations with LPC_PZ (−0.58) and LPC_FZ (−0.56). Female sad the strongest edge is between N2_FZ and N2_CZ (0.93). LPC_PZ has the highest expected influence centrality at 1.25. In the female sad condition’s behavioral-EEG network structure, the left node shows negative correlations with four edges: VPP_FZ (−0.61), N2_PZ (−0.58), LPC_FZ (−0.47), and N2_CZ (−0.42) (see Figure 5).

Across all four conditions, the strongest connections consistently occur between the frontal lobe and the central region. Reaction time (RT) does not exhibit significant correlations with different brain regions. In the happy and sad conditions, males display strong network stability, while females, particularly in the sad condition, exhibit more closely-knit and robust correlations.

## Discussion

The present study analyzed the dynamic processing of positive and negative body expressions in the brain using time-frequency analysis and further investigated gender differences in the processing of body expressions. Results revealed that individuals recognized positive body expressions more accurately than negative ones, which contradicts the “negative bias” theory (McRae and Gross, 2020) supported by previous research. This discrepancy may be due to the fact that studies related to “negative bias” often use expressions from negative conditions (e.g., fear) compared to neutral conditions as stimuli (Van den Stock et al., 2014; Hagenaars et al., 2014). Previous research has shown that negative emotions are often more difficult to identify than positive ones, and even in studies that have identified positive emotions, focusing only on behavioral data may lead to a ceiling effect that obscures group differences (Shriver et al., 2021; Walenda et al., 2021). This is consistent with the findings of this study. Additionally, females recognized body expressions more accurately than males, regardless of stimulus type, indicating that females have an advantage in processing physical emotional information. This supports the “attachment promotion” theory, which suggests that females are better at processing all categories of emotional information, enabling them, as nurturers, to respond more quickly and accurately to infants’ emotions, thus facilitating the establishment of secure attachments (Addabbo and Turati, 2020; Lambrecht et al., 2014).

The time-frequency analysis results are consistent with the time-domain results. When women process positive body expression, they activate higher power theta wave and lower alpha band than men. Theta band (4–7 Hz) waves were first observed in an individual’s sleep cycle, and were found to be related to the arousal level of the cerebral cortex, which is considered to be related to attention and memory search (Sorinas et al., 2020). The increase in Theta activity indicates that females have higher levels of cortical arousal in response to emotional information. There is an inverse relationship between Alpha band activity and cortical activation (Poláčková Šolcová and Lačev, 2017). In contrast, synchronized Alpha oscillations are usually associated with reduced cognitive activity (de Gelder et al., 2010). On the other hand, Alpha wave desynchronization is considered to be an indicator of cognitive demand, information processing, memory performance and attention (Irvin et al., 2022; Zhang et al., 2015). Thus, the time-frequency results similarly confirm the emotional processing advantage of women, who experience higher cortical activation than men when processing emotional information.

Based on the network analysis results, distinct neural mechanisms for recognizing bodily emotions in males and females are evident. Males consistently show the strongest connections between the frontal and central regions, with key ERP indicators such as N2_PZ and LPC_CZ having the highest influence centrality. This suggests crucial roles for these areas in processing emotions. Females, however, exhibit a more interconnected and robust network, especially in the sad condition, where the left node shows multiple negative correlations with other ERP indicators, reflecting more complex neural processing.

Reaction time (RT) did not significantly correlate with different brain regions, suggesting that neural processing of emotions operates independently of behavioral response times. Males displayed stable network connections, whereas females demonstrated a broader and more flexible network, indicating different neural strategies between genders. These findings emphasize the importance of gender-specific approaches in studying emotional processing and developing targeted interventions.

## Conclusion

There are some differences between males and females in the recognition of bodily emotions. Network analysis offers a more detailed perspective on their inherent interrelationships. However, these differences are not absolute and should not be overstated. Instead, we should emphasize individual variances and cultural backgrounds to gain a more comprehensive understanding of the nature of bodily emotion recognition abilities.

## Data Availability

The raw data supporting the conclusions of this article will be made available by the authors, without undue reservation.

## References

[ref1] Addabbo M. Turati C. (2020). Binding actions and emotions in the infant’s brain. Soc. Neurosci. 15, 470–476. doi: 10.1080/17470919.2020.1760130 32321361

[ref2] Atkinson A. P. Dittrich W. H. Gemmell A. J. Young A. W. (2004). Emotion perception from dynamic and static body expressions in point-light and full-light displays. Perception 33, 717–746. doi: 10.1068/p5096, PMID: 15330366

[ref3] Atkinson A. P. Tunstall M. L. Dittrich W. H. (2007). Evidence for distinct contributions of form and motion information to the recognition of emotions from body gestures. Cognition 104, 59–72. doi: 10.1016/j.cognition.2006.05.005 16831411

[ref4] Aviezer H. Trope Y. Todorov A. (2012). Body cues, not facial expressions, discriminate between intense positive and negative emotions. Science 338, 1225–1229. doi: 10.1126/science.1224313, PMID: 23197536

[ref5] Borsboom D. Cramer A. O. (2013). Network analysis: an integrative approach to the structure of psychopathology. Annu. Rev. Clin. Psychol. 9, 91–121. doi: 10.1146/annurev-clinpsy-050212-185608 23537483

[ref6] de Gelder B. Van den Stock J. Meeren H. K. Sinke C. B. Kret M. E. Tamietto M. (2010). Standing up for the body. Recent progress in uncovering the networks involved in the perception of bodies and bodily expressions. Neurosci. Biobehav. Rev. 34, 513–527. doi: 10.1016/j.neubiorev.2009.10.008, PMID: 19857515

[ref7] Epskamp S. Borsboom D. Fried E. I. (2018). Estimating psychological networks and their accuracy: a tutorial paper. Behav. Res. Methods 50, 195–212. doi: 10.3758/s13428-017-0862-1, PMID: 28342071 PMC5809547

[ref8] Epskamp S. Cramer A. O. Waldorp L. J. Schmittmann V. D. Borsboom D. (2012). qgraph: network visualizations of relationships in psychometric data. J. Stat. Softw. 48, 1–18. doi: 10.18637/jss.v048.i04

[ref9] Fruchterman J. Reingold M. (1991). Graph drawing by force-directed placement. Softw. Pract. Exp. 21, 1129–1164. doi: 10.1002/spe.4380211102

[ref10] Hagenaars M. A. Oitzl M. Roelofs K. (2014). Updating freeze: aligning animal and human research. Neurosci. Biobehav. Rev. 47, 165–176. doi: 10.1016/j.neubiorev.2014.07.021, PMID: 25108035

[ref11] Irvin K. M. Bell D. J. Steinley D. Bartholow B. D. (2022). The thrill of victory: savoring positive affect, psychophysiological reward processing, and symptoms of depression. Emotion 22, 1281–1293. doi: 10.1037/emo0000914 33252936 PMC8343962

[ref12] Lambrecht L. Kreifelts B. Wildgruber D. (2014). Gender differences in emotion recognition: impact of sensory modality and emotional category. Cogn. Emot. 28, 452–469. doi: 10.1080/02699931.2013.837378, PMID: 24151963

[ref13] Liang S. Liu C. Rotaru K. Li K. Wei X. Yuan S.. (2022). The relations between emotion regulation, depression and anxiety among medical staff during the late stage of COVID-19 pandemic: a network analysis. Psychiatry Res. 317:114863. doi: 10.1016/j.psychres.2022.114863, PMID: 36191555 PMC9509294

[ref14] Luo W. Feng W. He W. Wang N. Y. Luo Y. J. (2010). Three stages of facial expression processing: ERP study with rapid serial visual presentation. NeuroImage 49, 1857–1867. doi: 10.1016/j.neuroimage.2009.09.018, PMID: 19770052 PMC3794431

[ref15] Luo Y. Wu T. Gu R. (2012). Research progress on brain mechanism of emotion and cognition. J. Chin. Acad. Sci. 27, 31–41.

[ref16] McRae K. Gross J. J. (2020). Emotion regulation. Emotion 20, 1–9. doi: 10.1037/emo0000703 31961170

[ref17] Nagabhushan Kalburgi S. Kleinert T. Aryan D. Nash K. Schiller B. Koenig T. (2024). MICROSTATELAB: the EEGLAB toolbox for resting-state microstate analysis. Brain Topogr. 37, 621–645. doi: 10.1007/s10548-023-01003-5, PMID: 37697212 PMC11199309

[ref18] Peng K. Steele S. C. Becerra L. Borsook D. (2018). Brodmann area 10: collating, integrating and high level processing of nociception and pain. Prog. Neurobiol. 161, 1–22. doi: 10.1016/j.pneurobio.2017.11.004, PMID: 29199137 PMC5826795

[ref19] Pirau L. Lui F. (2023). “Frontal lobe syndrome” in StatPearls (Treasure Island, FL: StatPearls Publishing), 17. 30422576

[ref20] Poláčková Šolcová I. Lačev A. (2017). Differences in male and female subjective experience and physiological reactions to emotional stimuli. Int. J. Psychophysiol. 117, 75–82. doi: 10.1016/j.ijpsycho.2017.04.009, PMID: 28454989

[ref21] Rosenberg N. Ihme K. Lichev V. Sacher J. Rufer M. Grabe H. J.. (2020). Alexithymia and automatic processing of facial emotions: behavioral and neural findings. BMC Neurosci. 21:23. doi: 10.1186/s12868-020-00572-6, PMID: 32471365 PMC7257227

[ref22] Shriver L. H. Dollar J. M. Calkins S. D. Keane S. P. Shanahan L. Wideman L. (2021). Emotional eating in adolescence: effects of emotion regulation, weight status and negative body image. Nutrients 13:79. doi: 10.3390/nu13010079, PMID: 33383717 PMC7824438

[ref23] Sorinas J. Ferrández J. M. Fernandez E. (2020). Brain and body emotional responses: multimodal approximation for valence classification. Sensors 20:313. doi: 10.3390/s20010313, PMID: 31935909 PMC6982758

[ref24] Van den Stock J. Vandenbulcke M. Sinke C. B. de Gelder B. (2014). Affective scenes influence fear perception of individual body expressions. Hum. Brain Mapp. 35, 492–502. doi: 10.1002/hbm.22195, PMID: 23097235 PMC6869608

[ref25] Walenda A. Bogusz K. Kopera M. Jakubczyk A. Wojnar M. Kucharska K. (2021). Emotion regulation in binge eating disorder. Psychiatr. Pol. 55, 1433–1448. doi: 10.12740/PP/OnlineFirst/122212 35472237

[ref26] Zhang D. Zhao T. Liu Y. Chen Y. (2015). Comparison of facial expressions and body expressions: an event-related potential study. Acta Psychol. Sin. 47:963. doi: 10.3724/SP.J.1041.2015.00963

[ref27] Zieber N. Kangas A. Hock A. Bhatt R. S. (2014). Infants’ perception of emotion from body movements. Child Dev. 85, 675–684. doi: 10.1111/cdev.12134 23802842

[ref28] Zongbao L. Yun Y. Guangzhen Z. Yuankui Y. Wenming Z. Ruixin C. (2019). The recognition of bodily expression: an event-related potential study. Stud. Psychol. Behav. 17:318.

